# Comparison of Dry Eye and Corneal Sensitivity between Small Incision Lenticule Extraction and Femtosecond LASIK for Myopia

**DOI:** 10.1371/journal.pone.0077797

**Published:** 2013-10-29

**Authors:** Meiyan Li, Jing Zhao, Yang Shen, Tao Li, Li He, Hailin Xu, Yongfu Yu, Xingtao Zhou

**Affiliations:** 1 Department of Ophthalmology, Eye and Ear Nose and Throat Hospital of Fudan University, Myopia Key Lab of the Health Ministry, Shanghai, China; 2 Department of Biostatistics, School of Public Health of Fudan University, Shanghai, China; Zhongshan Ophthalmic Center, China

## Abstract

**Purpose:**

To investigate the changes in dry eye symptoms and clinical signs and corneal sensitivity after small incision lenticule extraction (SMILE) and femtosecond LASIK (femto-LASIK).

**Design:**

Prospective, non-randomized comparative study.

**Methods:**

The study included a total of 71 eyes of 71 patients; the SMILE group comprised 38 eyes of 38 patients, and the femto-LASIK group comprised 33 eyes of 33 patients. Ocular Surface Disease Index (OSDI), Tear film breakup time (TBUT), the Schirmer test without anesthesia (S1T), corneal fluorescein staining, and central corneal sensation were evaluated before surgery and at 1 week, 1 month, 3 months, and 6 months after surgery.

**Results:**

OSDI scores in both groups were increased immediately and returned to preoperative level at 1 month after surgeries. The TBUT values in both groups were reduced after surgeries relative to their preoperative scores. Patients in SMILE group were less likely to have corneal staining compared with those in the femto-LASIK group ([odds ratio] OR = 0.50, 95% [confidence interval] CI 0.28 to 0.93, *P* = 0.03). Central corneal sensitivity was decreased at all postoperative time points in both groups. However, the central corneal sensation scores in the SMILE group were greater than that in the femto-LASIK group at all of the postoperative time points (all *P*<0.05).

**Conclusions:**

SMILE surgeries resulted in a short-term increase in dry eye symptoms, tear film instability, and loss of corneal sensitivity. Furthermore, SMILE surgeries have superiority over femto-LASIK in lower risk of postoperative corneal staining and less reduction of corneal sensation.

## Introduction

Dry eye is a common complaint among patients who have undergone refractive surgeries, including laser in situ keratomileusis (LASIK), photorefractive keratectomy and femtosecond LASIK (femto-LASIK), and the incidence of dry eye varies among these patients. [1], [2], [3] It has been reported that patients who develop dry eye after refractive surgery also have elevated risks of developing subsequent refractive regression [4] and ocular surface damage. [5] In addition, refractive surgery procedures also interrupt the normal organization and regeneration of the corneal nerves, which in turn lead to a prolonged reduction in corneal sensation.

Great advances in the techniques used in refractive surgeries to correct myopia have been made from the LASIK technique to that of femtosecond lenticule extraction. It is believed that both the post-surgical development of dry eye and the decrease in corneal sensation are closely linked to the surgical amputation of the corneal nerve fibers that is produced by the creation of the flap during refractive surgery, regardless of the flap-cutting method that is used [4], [6], [7], [8], [9].

Small incision lenticule extraction (SMILE) is a novel all-in-one procedure that can be used in the surgical correction of myopia without the creation of a corneal flap. This technique makes it possible for SMILE patients to have lower risks of development of dry eye and decreased corneal sensation after surgery. A recent study [10] reported the changes in corneal sensation before and after SMILE surgery in a 3-month follow-up period. But there was no study that reported the changes in dry eye symptoms and dry eye signs before and after SMILE surgery. In the present article, we describe a prospective study to investigate the changes in post-surgical dry eye symptoms and clinical signs and corneal sensitivity following SMILE surgery for the treatment of myopia by comparing them with patients who had undergone femto-LASIK.

## Materials and Methods

### Ethics Statement

The Ethical Committee of the Fudan University EENT Hospital Review Board approved the study protocol, and the study was conducted in accordance with the principles of the Declaration of Helsinki regarding research involving human subjects. Each of the patients provided written informed consent to participate after the nature of the study had been explained to them. Both surgical treatments and involved examinations are standard clinical care. The surgeon (X.T.Z) made the decision to treat. Patients chose the treatments for themselves after the doctor had explained the nature of the two procedures.

### Subjects

In total, 71 consecutive patients who had undergone SMILE or femto-LASIK procedures for the treatment of myopia at the Fudan University Eye and ENT Hospital (Shanghai, People’s Republic of China) were recruited between June 2010 and February 2012. All of the patients underwent simultaneous SMILE or femto-LASIK procedures, and one eye of each patient was chosen at random for inclusion in the statistical analysis of the present study. The inclusion criteria for both procedures were an age of at least 18 years, a routine ophthalmic examination (with the exception of refractive error) with a stable refractive error and a minimum calculated residual corneal stromal bed thickness that was greater than 280 µm. Additionally, the spherical refractive errors should range from −3.00 diopter [D] to −10.00 D among myopia patients in the SMILE group, and these patients had astigmatisms of up to −5.00 D cyl. While in the femto-LASIK group, the patients should have spherical refractive errors of up to −11.00 D.

Patients with any one of the following conditions were excluded in either group: (1) those who used topical ocular medication on the day on which they participated in the study; (2) those who had external ocular diseases or had undergone ocular surgery during the last 6 months; or (3) those who had undergone permanent or temporary lacrimal punctum occlusion.

### Surgical Technique

The Carl Zeiss Meditec AG VisuMax femtosecond laser system was used to make surgical refractive corrections for patients in the SMILE group with a repetition rate of 500 kHz and a pulse energy of 130 nJ. The exact surgical procedure has been described previously by Sekundo et al. [11]. The intended thickness of the upper tissue arcade was 100 µm, and its intended diameter was 7.5 mm, which was 1 mm larger than the diameter of the refractive lenticule (6.5 mm). The side cuts made for access to the lenticule were set 90°apart at a width of 4.5 mm. The refractive lenticule of the intrastromal corneal tissue was dissected through the side-cut opening incision and was manually removed using forceps.

In the femto-LASIK group, flaps were created with a 500-kHz VisuMax femtosecond laser. The created flaps had diameters of 8.5 mm, standard 90° hinges and 90° side-cut angles. The lamellar and side cuts were achieved with energies of 185 nJ. The target flap thickness was 90 µm. The hinges were set in a superior orientation with a hinge length of 4.0 mm. Stromal tissue ablation was performed with the Mel-80 (Carl Zeiss Meditec) excimer laser using a tissue-saving function with a repetition rate of 250 kHz and a pulse energy of 150 nJ. The procedure was performed under topical anesthesia in all cases.

The same surgeon (X.T. Z) performed the operations on all of the patients who participated in the present study. Patients wore bandage soft contact lenses (ACUVE OASYS, Inc., FL, USA) until the next day of the operation. Postoperative topical medication regimens were identical for each eye and consisted of the administration of an ophthalmic solution of Levofloxacin 4 times per day for 7 days, a 0.1% fluorometholone solution 8 to 1 times per day with a taper over the course of 20 days, and a non-preservative tear supplement (Carboxymethylcellulose Sodium Eye Drops, Allergan, Inc., Irvine, CA) 4 times per day for 1 month.

### Ocular Surface Disease Index (OSDI) and Dry Eye Symptoms

The OSDI questionnaire was used to quantify the dry eye symptoms. Subjects were asked questions regarding the dry eye symptoms that they had experienced during a 1-week recall period; the OSDI questions were drawn from 3 different subscales: ocular symptoms, vision-related functions, and environmental triggers. Each answer was scored on a 4-point scale from zero (indicating no problems) to four (indicating a significant problem). Responses to all of the questions were combined to generate a composite OSDI score that ranged from 0 to 100, with higher OSDI scores indicating more severe symptoms. [12] Symptoms of dry eye, such as dryness, burning, foreign body sensation, stabbing pain, photophobia, and visual fluctuations, were also noted.

### Schirmer Test

The Schirmer test without anesthesia (S1T) for tear secretion function was performed by inserting a 30-mm Schirmer tear test strip (Jingming, Tianjing, China) into the inferior fornix at the junction of the middle and lateral thirds of the lower eyelid margin. Schirmer test strips remained in place for 5 minutes with the eyes closed. The extent of wetting was subsequently measured according to the scale provided by the manufacturer. Potential scores ranged from 0 to 30 mm, with lower scores indicating greater tear production abnormalities [13].

### Tear Breakup Time (TBUT)

Tear film stability was assessed based on TBUT. A fluorescein-impregnated strip (Jingming, Tianjing, China) that had been wetted with non-preservative saline solution was placed in the lower conjunctival sac, and the patient was asked to blink several times. [14] Using slit-lamp biomicroscopy with a cobalt blue filter, the time that elapsed before the first observation of tear film break-up after a complete blink was recorded as the TBUT. The test was repeated three times, and the average of the three measurements was calculated. Corneal fluorescein staining was graded as described by De Paiva et al. [15] The Schirmer test, TBUT test and corneal fluorescein staining test were performed by the same observer (J.Z) who was blind to the patient and postoperative time.

### Corneal Sensation Esthesiometer

Corneal sensation was measured with a Cochet-Bonnet esthesiometer (Luneau, Paris, France). This instrument consists of a nylon monofilament that is 60 mm in length and with diameter of 0.12 mm. The instrument was advanced perpendicular to the central surface of the cornea until contact between the instrument and the cornea was made. If the patient felt the filament, the response was defined positive. Corneal sensitivity was tested three times with each filament length, and the length of the filament was sequentially reduced from 60 mm in 5-mm steps. At least two positive responses among three attempts were considered a positive result at each filament length. The longest filament length that resulted in a positive result was considered the corneal threshold. All of the measurements were performed during slit-lamp examination by the same observer (M.Y.L) who was blind to the patient and postoperative time.

### Follow Up

The following parameters were evaluated in all of the patients before surgery and 1 week, 1 month, 3 months, and 6 months after surgery: TBUT, S1T, corneal fluorescein staining, and central corneal sensation. Dry eye symptoms were evaluated according to the OSDI questionnaire before surgery and 1 week, 1 month, 3 months, and 6 months after surgery.

### Statistical Analysis

Data were analyzed using SAS 9.3 statistical software (SAS Institute Inc., Cary, North Carolina, USA) and reported as means±standard deviation (SD). Comparisons of continuous variables were examined by independent Student’s t-test or Mann Whitney U test as appropriate, and a chi-square test was used for statistical analysis of categorical variables at the baseline. Taking preoperative SE at baseline as the selected covariate and different times for measurements as the repeated factor, Mixed Model was constructed to compare the data between the SMILE group and the femto-LASIK group for the following variables: OSDI, BUT, Schirmer scores, and central corneal sensitivity. A Generalized Estimating Equation (GEE) model was used to compare the percentage of corneal staining. In these models, we tested whether the variables were changed across the follow-up time in each group. In addition, we evaluated the mean differences between the SMILE group and the femto-LASIK group before surgery and at each postoperative time point for each variable. Bonferroni-corrected post hoc test was conducted to adjust the observed significant level for multiple comparisons. Statistical significance level was set at 0.05.

## Results

The number of eyes evaluated at each examination point is shown in Table 1. More than 90% of patients were followed up for 6 months. Table 2 shows the demographic data and preoperative characteristics of the patients in this study. In total, 38 eyes of 38 patients (10 males, 28 females) who received SMILE treatment were included, and 33 eyes of 33 patients (6 males, 27 females) who received femto-LASIK treatment were included. There were no statistically significant differences in age, sex, central corneal thicknesses, surgical ablation depths, or preoperative Corrected Distance Visual Acuity (CDVA) between the SMILE and femto-LASIK groups except with preoperative spherical equivalent (SE) and treated SE (*P = *0.009 and *P = *0.008, respectively).

**Table 1:** Number of eyes evaluated at each examination point.

Groups	Preoperatively	Postoperatively			
		1 week	1 month	3 months	6 months
SMILE	38 (100%)	37 (97%)	37 (97%)	36 (95%)	36 (95%)
femto-LASIK	33 (100%)	32 (97%)	31 (94%)	30 (91%)	30 (91%)

SMILE: small incision lenticule extraction. femto-LASIK: femtosecond LASIK.

**Table 2:** Demographic data and preoperative characteristics of the study participants.

Characteristics	SMILE group (n = 38)	femto-LASIK group (n = 33)	*P* value
Age (mean±SD, years)	28.21±7.04	27.33±6.58	0.59*
Male/Female	10/28	6/27	0.41†
Preoperative SE (D)			
Mean	−6.68±1.34	−7.96±2.61	0.009*
Range	−9.00 to −4.25	−12.50 to −2.00	
Treated SE (D)			
Mean	−7.15±1.49	−8.55±2.84	0.008*
Range	−9.75 to −4.00	−13.38 to−2.50	
Corneal thickness (µm)	544.74±30.27	538.52±27.69	0.44*
Ablation depth (mean±SD, µm)	131.46±22.96	133.24±30.45	0.45*
Range	77 to 165	51 to 168	
CDVA (logMAR)	−0.02±0.05	−0.00±0.07	0.33*

SMILE: small incision lenticule extraction. femto-LASIK: femtosecond LASIK.

SD: standard deviation. SE: spherical equivalent. D: diopter. CDVA: corrected distance visual acuity.

* independent Student’s t-test or Mann Whitney U test.

† chi-square test.

At postoperative 6 months, the Uncorrected Distance Visual Acuity (UDVA) and CDVA was −0.08±0.10 logMAR (Snellen equivalent, 20/16.6), −0.08±0.06 logMAR (Snellen equivalent, 20/16.6) in SMILE group and −0.04±0.10 logMAR (Snellen equivalent, 20/18), −0.07±0.07 logMAR (Snellen equivalent, 20/17) in femto-LASIK group. There was no difference between the two groups in the postoperative UDVA (*P = *0.15), CDVA (*P = *0.90).

**Figure 1:**
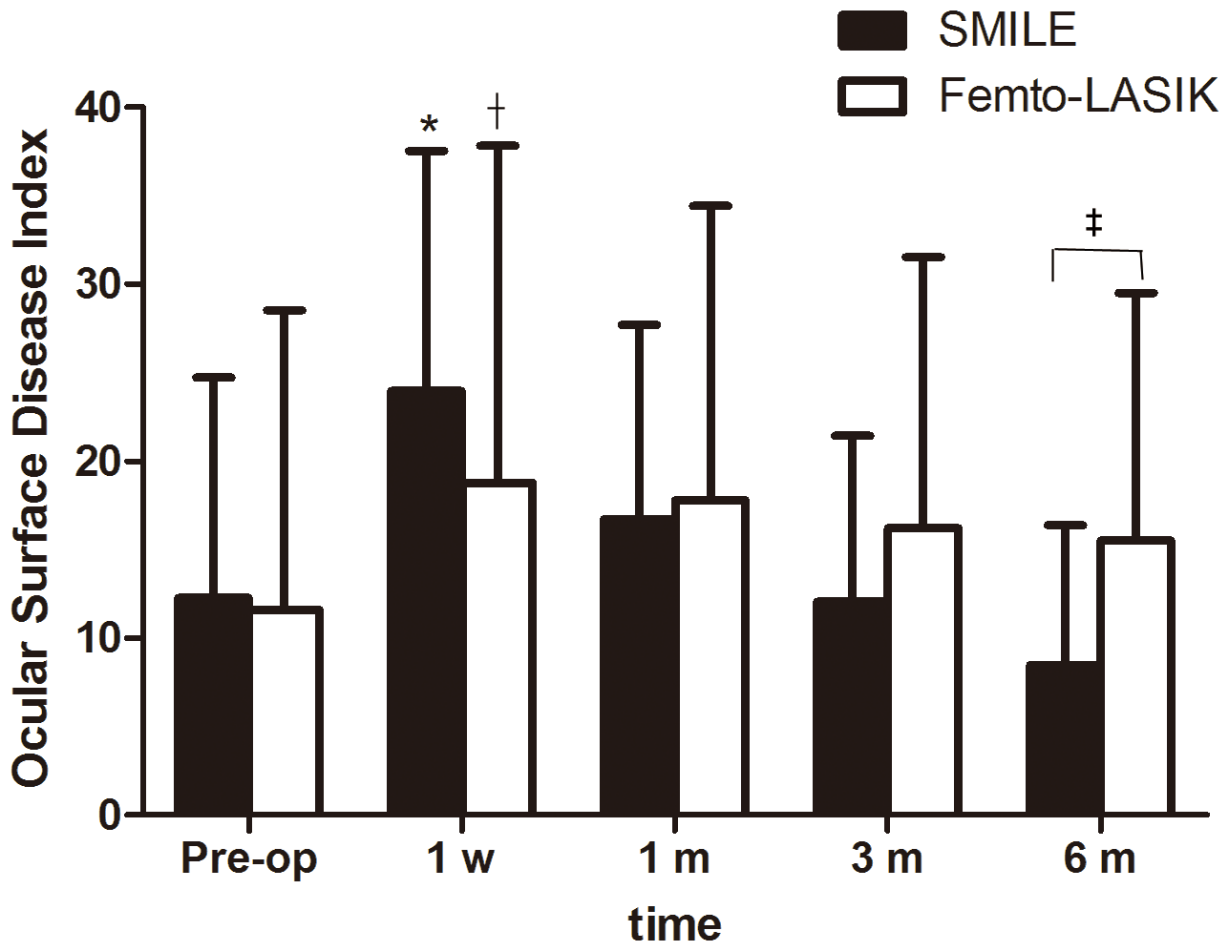
Time dependent changes in the Ocular Surface Disease Index (OSDI) (mean ± SD) after small incision lenticule extraction (SMILE) and femtosecond LASIK (femto-LASIK). *Significantly higher than the preoperative level (SMILE; *P*<0.05). ^†^Significantly higher than the preoperative level (femto-LASIK; *P*<0.05).^ ‡^Significantly different between groups (*P*<0.05).

The mean preoperative OSDI scores were 12.26±12.45 in the SMILE group and 11.89±16.92 in the femto-LASIK group, and there was no significant difference between the two groups (*P = *0.42). There were statistically significant increases in the postoperative OSDI scores in both SMILE group and femto-LASIK groups at 1 week (*P*<0.0001 and *P = *0.01, respectively) compared with preoperative values, but returned to preoperative level at 1 month (*P = *0.06 and *P = *0.05, respectively). When the SMILE group was compared with the femto-LASIK group, no statistically significant difference was found at any time point (*P = *0.14, *P = *0.95, and *P = *0.18 for 1 week, 1 month, and 3 months respectively) except with postoperative 6 months (P = 0.02). (Figure 1 and Table 3).

**Table 3:** Preoperative and postoperative clinical parameters.

	Pre-op	1 week	1 month	3 months	6 months
Ocular Surface Disease Index (mean±SD)					
SMILE	12.26±12.45	23.95±13.54	16.72±10.96	12.05±9.38	8.47±7.89
femto-LASIK	11.59±16.92	18.78±19.01	17.77±16.64	16.22±15.29	15.50±14.00
Tear Breakup Time (mean±SD, s)					
SMILE	8.58±4.42	4.32±3.57	5.68±4.84	5.03±3.83	7.06±3.85
femto-LASIK	7.88±5.57	4.70±3.65	3.77±2.91	4.43±4.22	4.97±3.57
Schirmer Scores (mean±SD, mm)					
SMILE	14.63±7.51	13.51±10.96	12.11±7.58	14.14±9.38	13.28±8.72
femto-LASIK	15.36±9.47	10.00±8.28	10.90±7.99	13.73±9.54	13.17±9.32
Corneal Sensation (mean±SD, mm)					
SMILE	58.16±3.37	29.59±17.73	30.00±16.37	37.92±15.42	46.94±11.73
femto-LASIK	57.27±6.26	20.61±15.50	21.45±15.34	27.50±17.46	39.17±16.09

SMILE: small incision lenticule extraction. femto-LASIK: femtosecond LASIK.

SD: standard deviation.

**Figure 2:**
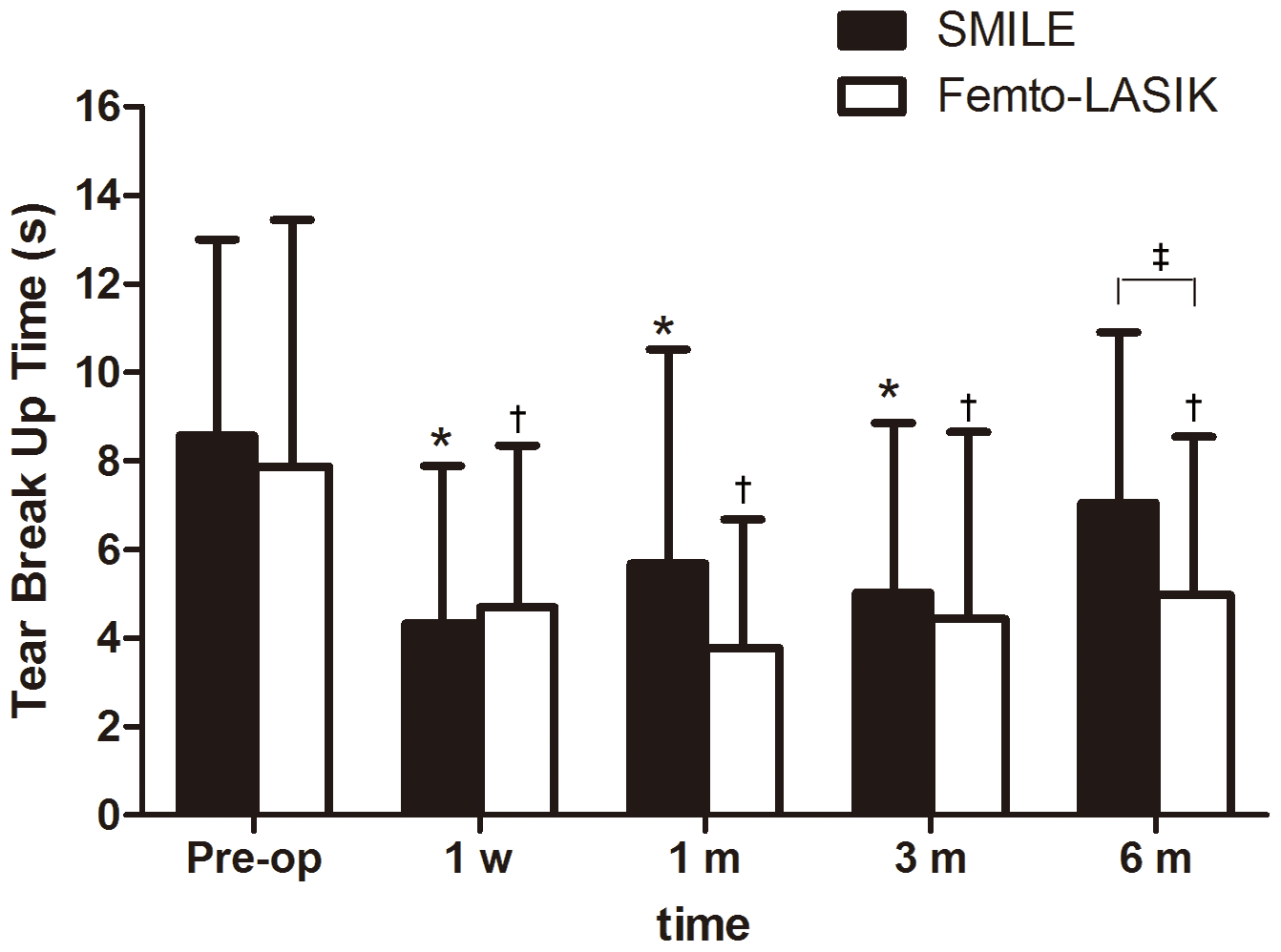
Time dependent changes in the tear breakup time (TBUT) scores (mean ± SD, s) after small incision lenticule extraction (SMILE) and femtosecond LASIK (femto-LASIK). *Significantly shorter than the preoperative level (SMILE; *P*<0.05).^ †^Significantly shorter than the preoperative level (femto-LASIK; *P*<0.05).^ ‡^Significantly different between groups (*P*<0.05).

Figure 2 shows the mean TBUT. Statistically significant decreases in the TBUT of the SMILE group were found at 1 week, 1 month, and 3 months after surgery compared with the preoperative TBUT values (*P*<0.0001, *P = *0.001, and *P*<0.0001 respectively), but returned to preoperative level at postoperative 6 months (*P = *0.08). In the femto-LASIK group, statistically significant decreases in TBUT was found at any time point (*P = *0.0007, *P*<0.0001, *P = *0.0003, and *P = *0.002 for 1 week, 1 month, 3 months, and 6 months respectively) after surgery. In addition, significant differences in the mean TBUT scores between the SMILE and femto-LASIK groups were found at postoperative 6 months (*P = *0.03). (Table 3).

**Figure 3:**
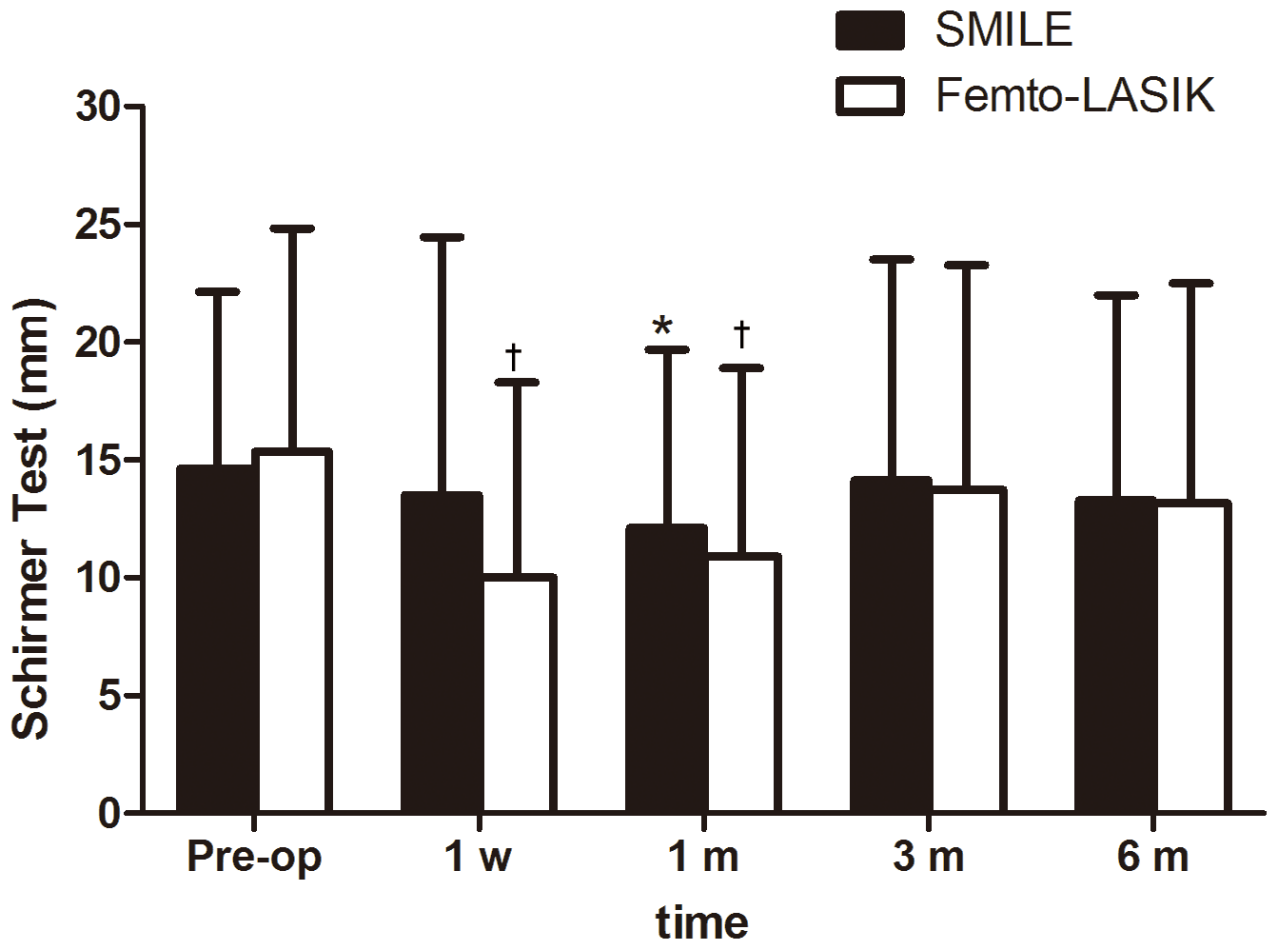
Time dependent changes in the Schirmer test results (mean ± SD, mm) after small incision lenticule extraction (SMILE) and femtosecond LASIK (femto-LASIK). *Significantly shorter than the preoperative level (SMILE; *P*<0.05). ^†^Significantly shorter than the preoperative level (femto-LASIK; *P*<0.05).

The results of the Schirmer test showed that in the SMILE group there were no statistically significant decreases in S1T values at any of the postoperative time points compared with the baseline (all *P* values were >0.05) except with postoperative 1 month (*P = *0.04). In the femto-LASIK group, mean S1T value was significantly decreased at the 1-week and 1-month postoperative visit (*P = *0.003 and *P = *0.003, respectively). At 3-month, there was no significant difference in mean S1T value from preoperative levels. (Figure 3) No statistical difference was found between the two groups at all postoperative time intervals. (Table 3).

Because of the interaction of group and time in GEE was not statistically significant (*P = *0.80), GEE model without interaction was fitted. The fitting results depicted that there was no significant difference between the two groups (SMILE vs. femto-LASIK) in the percentage of patients with preoperative corneal fluorescein staining (*P = *0.14). The percentage of corneal staining was significantly greater at 1-week (*P = *0.007) and 1- month (*P = *0.04) visits from preoperative levels. At 3 months (*P = *0.81), 6 months (*P = *0.06), there was no significant difference in the percentage of corneal staining from preoperative levels. Patients in SMILE group were less likely to have corneal staining compared with those in the femto-LASIK group ([odds ratio] OR = 0.50, 95% [confidence interval] CI: 0.28 to 0.93, *P = *0.03).

**Figure 4:**
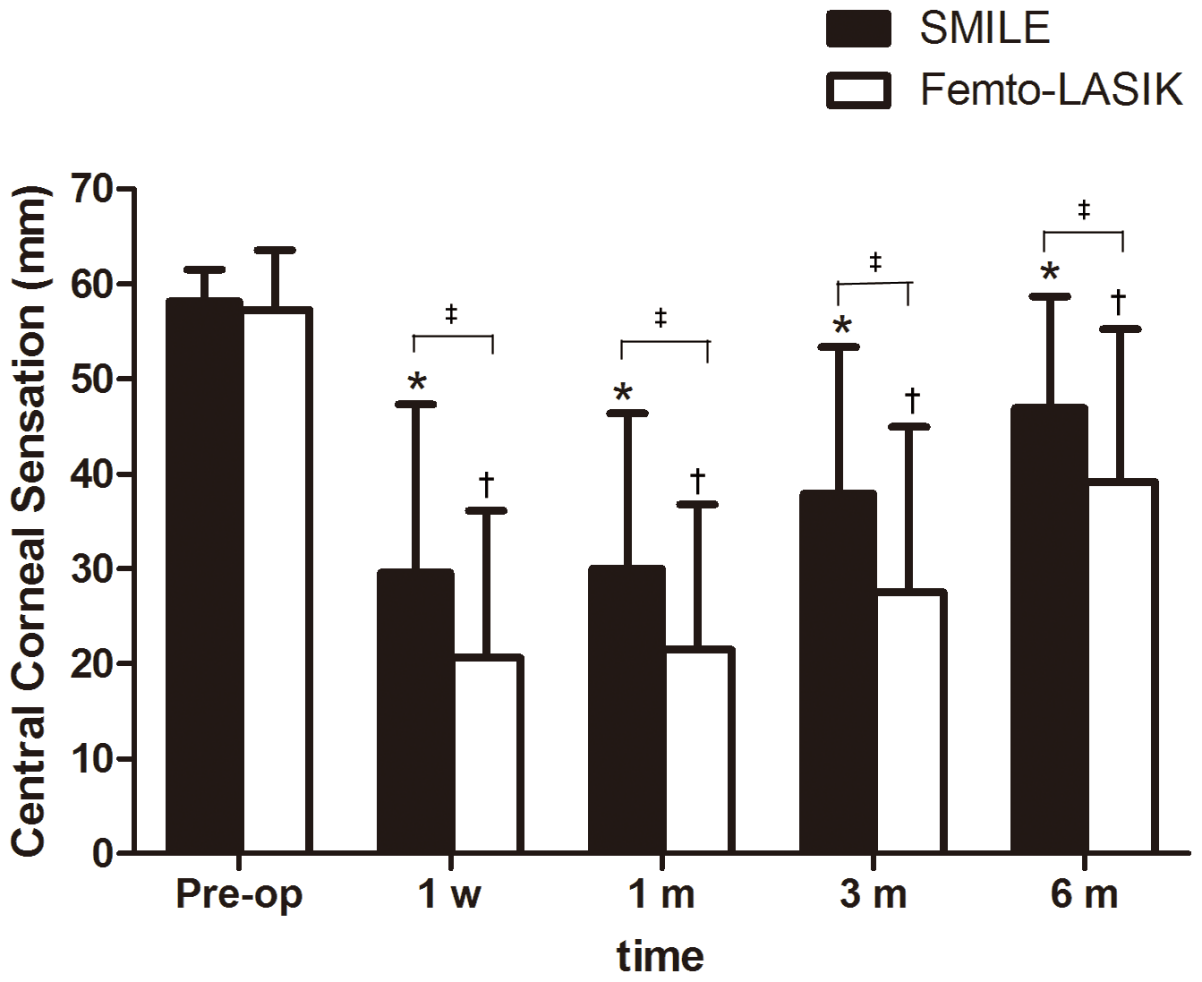
Time dependent changes in the mean central corneal sensations (mean ± SD, mm) after small incision lenticule extraction (SMILE) and femtosecond LASIK (femto-LASIK). *Significantly lower than the preoperative level (SMILE; *P*<0.05). ^†^Significantly lower than the preoperative level (femto-LASIK; *P*<0.05).^ ‡^Significantly different between groups (*P*<0.05).

Compared with the preoperative measurements, central corneal sensitivity in both groups were decreased at 1 week (*P*<0.0001), 1 month (*P*<0.0001), 3 months (*P*<0.0001), and 6 months (*P*<0.0001) after surgery. Although there was no statistically significant difference in the central corneal sensitivities between the SMILE and femto-LASIK groups at the preoperative time point (*P* = 0.45), the mean central corneal sensitivity in the SMILE group was greater than that in the femto-LASIK group at all of the postoperative time points (*P = *0.03, *P = *0.04, *P = *0.01, and *P = *0.03 for 1week, 1 month, 3 months, and 6 months after surgery, respectively). In other words, the observed reductions in central corneal sensation were less pronounced in patients who had received SMILE treatment than in those who had undergone femto-LASIK treatment (Figure 4 and Table 3).

## Discussion

Both dry eye symptoms and corneal sensation reduction are common after all types of corneal refractive surgeries. Dry eye could negatively affect the daily activities of patients, and the morbidity of dry eye increases with symptom severity. [16] In the current study, we observed time-dependent changes in the measurements of dry eye symptoms and clinical signs and central corneal sensation before and after SMILE surgery, and we compared these outcome measures with the same outcome measures in a group of patients who had undergone femto-LASIK treatment.

In the current study, we observed a postoperative reduction in central corneal sensitivity of patients in both SMILE group and femto-LASIK group compared with their preoperative sensitivity measurements. In addition, there was a trend toward increase of corneal sensitivity after surgery. The mean central corneal sensation in the SMILE group was greater than that in the femto-LASIK group at all of the postoperative time points, which indicated that deterioration of corneal sensation was greater after femto-LASIK than after SMILE. Our results were partly consistent with what Wei *et al*. [10] found. They found that the corneal sensitivity after SMILE surgery was better than that after SMILE surgery at all postoperative visits (1 week, 1 month and 3 months). But they found that at postoperative 3 months after SMILE surgery, the corneal sensitivity had recovered to preoperative level, which was not noted in present study. At present, we could not determine the reason for this discrepancy, thus further studies are needed to elucidate this point.

The preponderance of literature supports the hypothesis that the most important factor in the pathophysiology of refractive surgery-induced dry eye and decreased corneal sensitivity is the transection of corneal nerves in the anterior third of the corneal stroma that occurs during the surgeries. [17] The amputation of corneal nerves that occurs during refractive surgeries may subsequently result in the suppression of tear secretion from the lacrimal gland, mucin expression on the corneal epithelium, and frequent blinking because these homeostasis-maintaining behaviors are driven by a neuronal feedback loop that is mediated by corneal sensitivity. [18], [19] Therefore, the most significant reason underlying better corneal sensitivity after the SMILE procedure is less amputation of corneal nerves, which avoids the process of lifting a flap by extracting a lenticule from the stroma via a 3.0–5.0-mm incision, allowing less severing of corneal nerve fibers, whereas the femto-LASIK procedure requires the severing of all the superficial corneal nerves except the nerves that are located at the position of the hinge.

The post-surgical OSDI scores of both the SMILE and femto-LASIK groups significantly increased relative to the corresponding preoperative scores in a certain period of time after surgery. Previous studies have reported dry eye parameters following femto-LASIK surgeries that are consistent with the results of our study. [1], [20] In addition, this study found that the OSDI scores of both groups returned to preoperative level by 1 month after surgery, indicating that patients in both groups could recover quickly from subjective dry eye symptoms. There is one thing to note that at postoperative 6-month visit, the OSDI scores in the femto-LASIK group were statistically higher than that in the SMILE group. But we could not come to a conclusion that the subjective dry eye symptoms at postoperative 6 months in SMILE-treated eyes were less severe than those in femto-LASIK-treated eyes, because there were no statistically significant differences in the mean scores at 6-month visit compared with respective preoperative scores in both groups.

Although the postoperative tear secretion showed a transient decrease in both groups, there was no significant difference between the two groups. At present, the notion regarding the post-surgical effects of femto-LASIK on tear secretion function has not reached a consensus. Mian *et al.* [20] and Golas *et al.* [21] reported that the tear secretion function was suppressed during the first 3 months following the surgical procedure and returned to normal levels by 6 months after surgery, while Horwath-Winter *et al*. [22] reported that there were no significant changes in Schirmer test results. Toda I *et al.* [7] thought that the decrease in tear secretion was involved in the development of dry eye symptoms after LASIK and they found that the tear function after LASIK was parallel to that of corneal sensitivity. However, this parallel relationship was not observed in the present study. There is one thing to note from Figure 3 that the S1T values in the SMILE group showed a statistically significant decrease at 1-month visit, which is out of expectation in that it did not show the greatest decrease at 1-week visit as femto-LASIK did. The possible reason for this finding may be the different corneal wounding healing process in SMILE-treated eyes from femto-LASIK-treated eyes. Further studies with in vivo confocal microscopy may be needed to investigate the difference in cornea changes between the two groups.

Our study also found that patients receiving the SMILE procedure were significantly less likely to have corneal fluorescein staining than patients receiving the femto-LASIK procedure (odds ratio of 0.50). Wilson et al. [9], [17] reported LASIK-induced neurotrophic epitheliopathy with punctate epitheliopathy and suggested that this may be attributable to loss of trophic influence from severed corneal nerve trunks. Although the epithelial-stromal-neural-lacrimal gland-immune cellular interactions are interwoven in the corneal response to injury after refractive surgery, the results of in vitro co-culture studies suggested that neurons and corneal epithelial cells support one another trophically through the mutual release of soluble factors. [23] Therefore, the lower risk in developing corneal fluorescein staining in SMILE-treated eyes may be attributable to less cornea nerves damage in SMILE procedure relative to femto-LASIK procedure.

Based on a comprehensive analysis of OSDI, TBUT and corneal sensation in SMILE group, we could find that dry eye symptoms (OSDI scores), returned to preoperative level by postoperative 1 month, with TBUT and corneal sensitivity testing not showing dramatic improvements, but tear resection. Another concern is that although SMILE-treated eyes had less reduction in corneal sensitivity than femto-LASIK treated eyes, the SMILE treated eyes did not show obvious superiority in dry eye symptoms and signs. One possible explanation for this discrepancy is the lack of correlations between patients’ irritative ocular symptoms and the results of clinical tests for dry eye (BUT, S1T), [24], [25] as well as corneal sensitivity, [26] although it is considered that the post-surgical development of dry eye is closely linked to the surgical amputation of the corneal nerve fibers [4], [6], [7], [8], [9].

There are two limitations to the present study. We did not employ a randomized design. Although the preoperative characteristics were similar between the two groups, the surgical methods could not be randomly assigned to patients. In addition, the cap/flap sizes and anterior cut between the two procedures were not the same, but these differences would be unlikely to be a significant factor contributing to the significant difference in study results between the two groups.

This study adds an important issue that SMILE can make less effect on corneal sensitivity. But SMILE surgery has its own limitations that it can not correct the spherical refractive errors greater than −10.0 D and can not do enhancement procedure by now. Other aspects such as postoperative high-order aberrations and corneal topography changes need further studied to compare with the established femto- LASIK to gain a full scope of information on SMILE.

In conclusion, the results of this study indicate that SMILE surgery could result in dry eye symptoms, tear film instability, and decreased corneal sensitivity. Furthermore, SMILE has superiority over femto-LASIK in lower risk of postoperative corneal fluorescein staining and less reduction of corneal sensation.
